# Biochemical changes associated with non-alcoholic fatty liver disease in response to berberine treatment: a systematic review and meta-analysis of clinical and preclinical research

**DOI:** 10.3389/fphar.2025.1460643

**Published:** 2025-08-29

**Authors:** Wenyu Zhu, Lele Yang, Yufan Dai, Hanyang Wang, Jiaxuan Zhou, Lina Xia, Tao Shen

**Affiliations:** ^1^ School of Health Preservation and Rehabilitation, Chengdu University of Traditional Chinese Medicine, Chengdu, China; ^2^ School of Clinical Medicine, Chengdu University of Traditional Chinese Medicine, Chengdu, China; ^3^ School of Basic Medicine, Chengdu University of Traditional Chinese Medicine, Chengdu, China; ^4^ Siemens PLM Software, Chengdu, China

**Keywords:** non-alcoholic fatty liver disease, berberine, clinical study, preclinical research, meta-analysis

## Abstract

**Background:**

Non-alcoholic fatty liver disease (NAFLD) represents a global health challenge. Berberine, an isoquinoline alkaloid traditionally used for metabolic disorders, has garnered attention for its potential therapeutic interventions.

**Objective:**

To comprehensively review and perform a meta-analysis of berberine’s effects on NAFLD across clinical and preclinical studies.

**Methods:**

A comprehensive literature search was conducted across five databases from their inception to May 2024. We included randomized controlled trials and animal studies that evaluated berberine’s impact on NAFLD using specified biochemical markers.

**Results:**

Out of 487 screened studies, 22 (4 clinical and 18 preclinical) were included. Clinically, berberine significantly reduced fasting blood glucose (FBG) levels, with an effect size of 0.53 (95% CI: 0.04–1.01). In preclinical settings, berberine consistently demonstrated benefits across several markers, including alanine aminotransferase (ALT), aspartate aminotransferase (AST), and lipid profiles, despite significant heterogeneity in some outcomes.

**Conclusion:**

Berberine presents promising therapeutic avenues for NAFLD management, especially in terms of glucose metabolism. Further rigorous, well-designed trials are needed to substantiate these findings.

**Systematic Review Registration:**

https://www.crd.york.ac.uk/prospero/, Identifier CRD42023459618.

## 1 Introduction

Non-alcoholic fatty liver disease (NAFLD), now increasingly referred to as Metabolic Associated Steatohepatitis (MASLD),despite the recent nomenclature shift to MASLD, this systematic review and meta-analysis will continue to use NAFLD to align with the studies included and the existing literature.NAFLD is characterized by the accumulation of triglycerides in more than 5% of hepatocytes, occurring without significant alcohol consumption (Powell et al., 2021). The disease spectrum ranges from steatosis, often accompanied by mild inflammation (NAFL), to a more inflammatory subtype known as non-alcoholic steatohepatitis (Friedman et al., 2018). The condition is a burgeoning global health concern, affecting approximately one-quarter of the world’s population (Younossi et al., 2018). Emerging research highlights NAFLD’s systemic consequences, extending beyond liver-centric complications like cirrhosis and hepatocellular carcinoma to cardiovascular disease and metabolic syndromes (Byrne and Targher, 2015). Despite the increasing prevalence and systemic impact of MASLD, there are presently limited pharmacological therapies approved by regulators. Notably, the FDA has recently fast-tracked the approval of Resmetirom for the treatment of MASLD, marking a significant advancement in therapeutic options for patients (Noureddin et al., 2024). However, further research is needed to evaluate the long-term effects and applicability of Resmetirom across diverse patient populations. Current treatment strategies focus mainly on modifying risk factors such as obesity and insulin resistance through lifestyle interventions (Younossi et al., 2018). Nonetheless, the efficacy of these non-pharmacological approaches varies widely among individuals, influenced by factors including patient compliance, genetic predispositions, and underlying comorbidities (Rinella and Sanyal, 2016).

Given the increasing prevalence and limited treatment options, there is heightened attention on alternative and complementary medical therapies. One such candidate is berberine, an isoquinoline alkaloid with a longstanding tradition in traditional Chinese medicine, primarily used to treat various gastrointestinal conditions (Habtemariam, 2020). Interestingly, recent scientific investigations have started exploring berberine’s potential applications beyond gastrointestinal disorders, particularly its role in alleviating symptoms of NAFLD (Feng et al., 2019).

Current empirical findings regarding berberine’s efficacy in the treatment of NAFLD are inconsistent but promising. Yan et al., for instance, found that berberine supplementation in conjunction with lifestyle interventions led to significant reductions in hepatic fat content and improvements in various metabolic markers (Yan et al., 2015). Likewise, Chang et al. demonstrated that berberine induced significant alterations in serum lipid profiles, including a substantial reduction in ceramide and ceramide-1-phosphate levels when compared to lifestyle interventions alone (Chang et al., 2016). On another research front, Wu et al. reported that berberine positively influenced brown adipose tissue development and thermogenic activity, while Yu et al. attributed its efficacy to specific molecular mechanisms, such as reducing mitochondrial oxygen consumption rates and ATP synthesis (Wu et al., 2019; Yu et al., 2021).

To enhance the rationale for focusing on NAFLD, it is crucial to discuss the specific metabolic and inflammatory pathways that berberine may influence. Recent literature has highlighted berberine’s antioxidant and anti-inflammatory mechanisms, which are particularly relevant to NAFLD pathogenesis. A systematic review by Nahid Jivad et al. (2024) discussed the anti-inflammatory and antioxidant mechanisms of berberine, showing its neuroprotective properties by reducing oxidative stress, neuroinflammation, and anti-apoptosis effects. It also increases brain-derived neurotrophic factor (BDNF) release and reduces transforming growth factor-beta (TGF-β1) and hypoxia-inducible factor 1α (HIF-1α) (Jivad et al., 2024; Cai et al., 2021).Furthermore, berberine increases scavenging of reactive oxygen species (ROS), activates nuclear factor erythroid 2-related factor 2 (Nrf2), endogenous antioxidant enzymes, heme oxygenase-1 (HO-1), and inhibits lipid peroxidation, inserting its antioxidant activity. Additionally, berberine shows anti-inflammatory activity by reducing Interleukin (IL)-1β, IL-6, and tumor necrosis factor-alpha (TNF-α) levels and through inhibiting cyclooxygenase-2 (COX-2), and including nuclear factor κB (NF-κB) (Jivad et al., 2024; Aghili et al., 2024; Wang et al., 2024; Shakeri et al., 2024).

These mechanisms suggest that berberine could potentially modulate key pathways involved in NAFLD, such as the Nrf2/HO-1 antioxidant pathway and the NF-κB-mediated inflammatory response. By integrating these recent findings, our manuscript provides a more comprehensive understanding of berberine’s mechanisms, supporting its potential therapeutic role in NAFLD and other diseases.

Current clinical and preclinical data collectively suggest the potential benefits of berberine in NAFLD, but the conclusions remain inconsistent due to small sample sizes, inconsistent outcome measures, and population heterogeneity. More importantly, previous systematic reviews have either focused solely on clinical outcomes or only integrated animal experiments, lacking a cross-species evidence chain. This study is the first to conduct a paired meta-analysis of human RCTs and animal models within the same framework, systematically evaluating the effects of berberine on liver biochemistry, non-invasive fibrosis markers, and metabolic parameters in NAFLD. Further dose-response and mechanism subgroup analyses are also conducted to provide precise evidence for its clinical translation. We believe that this comprehensive strategy can assist clinical decision-makers in assessing the role of berberine in the future management of NAFLD.

## 2 Materials and methods

### 2.1 Protocol

This systematic evaluation and meta-synthesis were conducted in strict adherence to the guidelines set out in the PROSPERO registration (CRD42023459618).

### 2.2 Search strategy

In this study, researchers conducted a comprehensive literature search across five key databases: PubMed, EMBASE, Cochrane Central Register of Controlled Trials, Ovid, and Web of Science, covering the period from the establishment of each database up to May 2024. The search strategy was crafted based on the PICOS framework, where: (P) Population included both human and animal subjects diagnosed with NAFLD; (I) Intervention constituted the use of berberine; (C) Comparators involved a control group that received standard care or underwent lifestyle modifications; (O) Outcomes were assessed through a variety of biochemical markers related to NAFLD; and (S) Study types included clinical trials and pre-clinical studies. For clarity, a comprehensive search strategy utilized in the PubMed database is outlined in Table 1.

**TABLE 1:** Search strategy for PubMed.

Search	PUBMED
#1	((((((((((((Non-alcoholic Fatty Liver Disease [MeSH Terms]) OR (Non alcoholic Fatty Liver Disease [Title/Abstract])) OR (NAFLD [Title/Abstract])) OR (Nonalcoholic Fatty Liver Disease [Title/Abstract])) OR (Fatty Liver, Nonalcoholic [Title/Abstract])) OR (Fatty Livers, Nonalcoholic [Title/Abstract])) OR (Liver, Nonalcoholic Fatty [Title/Abstract])) OR (Livers, Nonalcoholic Fatty [Title/Abstract])) OR (Nonalcoholic Fatty Liver [Title/Abstract])) OR (Nonalcoholic Fatty Livers [Title/Abstract])) OR (Nonalcoholic Steatohepatitis [Title/Abstract])) OR (Steatohepatitides, Nonalcoholic [Title/Abstract])) OR (Steatohepatitis, Nonalcoholic [Title/Abstract])
#2	((Berberine [MeSH Terms]) OR (Berberine [Title/Abstract]) OR (Umbellatine [Title/Abstract]))
#3	#1 AND #2
#4	randomzied controlled trials [Publication Type]
#5	#3 AND #4

### 2.3 Inclusion criteria

1) Experimental groups received berberine-based therapeutic interventions targeting both human and animal subjects diagnosed with NAFLD. 2) Control groups predominantly consisted of individuals who either did not receive berberine treatment or were subjected solely to lifestyle modification approaches. 3) Methodologies employed included both randomized clinical trials and animal model studies. 4) For outcome measurement, each study had to report on at least one of the following metrics: total cholesterol (TC), hepatic TC, liver TC, triglyceride (TG), hepatic TG, liver TG, low-density lipoprotein (LDL), high-density lipoprotein (HDL), alanine aminotransferase (ALT), aspartate aminotransferase (AST), body mass index (BMI), fasting blood glucose (FBG), fasting insulin (FINS), free fatty acid (FFA), hepatic weight, and liver-to-body weight ratio.

### 2.4 Exclusion criteria

1) Studies with incomplete datasets or insufficient data disclosure, and 2) investigations from non-randomized controlled experiments, including quasi-randomized controlled trials, study protocols, conference abstracts, case studies, or scholarly correspondence.

### 2.5 Study selection

Utilizing the bibliographic software EndNote for literature management, two investigators initially scanned the article titles to eliminate duplicates, non-randomized controlled trials, review articles, conference proceedings, study protocols, and correspondences. Subsequently, the abstracts were reviewed by both investigators to ascertain which studies should be considered for inclusion or exclusion. Finally, the residual articles were comprehensively read by both investigators to determine their eligibility for inclusion. Throughout this procedure, each investigator independently evaluated the articles, and then a final comparison of the selected literature was made. In instances where the selections diverged, a third investigator intervened to facilitate discussion and arrive at a consensus.

### 2.6 Data extraction

A structured data collection form comprising seven predefined elements was employed to capture pertinent information for study inclusion, categorized under the following parameters: 1) author, 2) year of publication, 3) country of study, 4) study period, 5) sample size, 6) mean age for clinical trials or body weight for preclinical studies, and 7) details of the intervention involving berberine.

### 2.7 Risk of bias of individual studies

Two investigators carried out an autonomous evaluation of bias risk, adhering to the guidelines set forth by the Cochrane Handbook for Systematic Reviews of Interventions, version 5.1.0, specifically to assess bias risk in Randomized Controlled Trials. Seven key aspects were scrutinized for this purpose: 1) randomized sequence generation, 2) treatment allocation concealment, 3) blinding of participants, 4) blinding of personnel, 5) incomplete outcome data, 6) selective reporting, and 7) other sources of bias. Based on the count of elements with a high likelihood of inducing bias, each trial was classified into one of three risk categories: high risk (five or more elements), moderate risk (three to four elements), and low risk (fewer than three elements) (Higgins et al., 2011).

### 2.8 Data analysis

In research contexts, when berberine is used as the primary intervention, all measured variables are treated as continuous and presented as means accompanied by their standard deviations (SD). In the present analysis, continuous variables will be articulated either as the mean difference (MD, representing the absolute discrepancy between the average outcomes of the treatment and control cohorts, determined on an identical scale) or the standardized mean difference (SMD, representing the variation in outcomes between groups normalized by the outcome’s standard deviation among subjects, applicable when merging data from studies employing disparate scales). These results will be framed within 95% confidence intervals (CI) for subsequent analytical scrutiny. Acknowledging the inherent variability across individual studies, we will employ a random-effects model if the I^2^ statistic exceeds 50%; conversely, a fixed-effects model will be used when the I^2^ value is below 50%.

## 3 Results

### 3.1 Search outcomes

**FIGURE 1:**
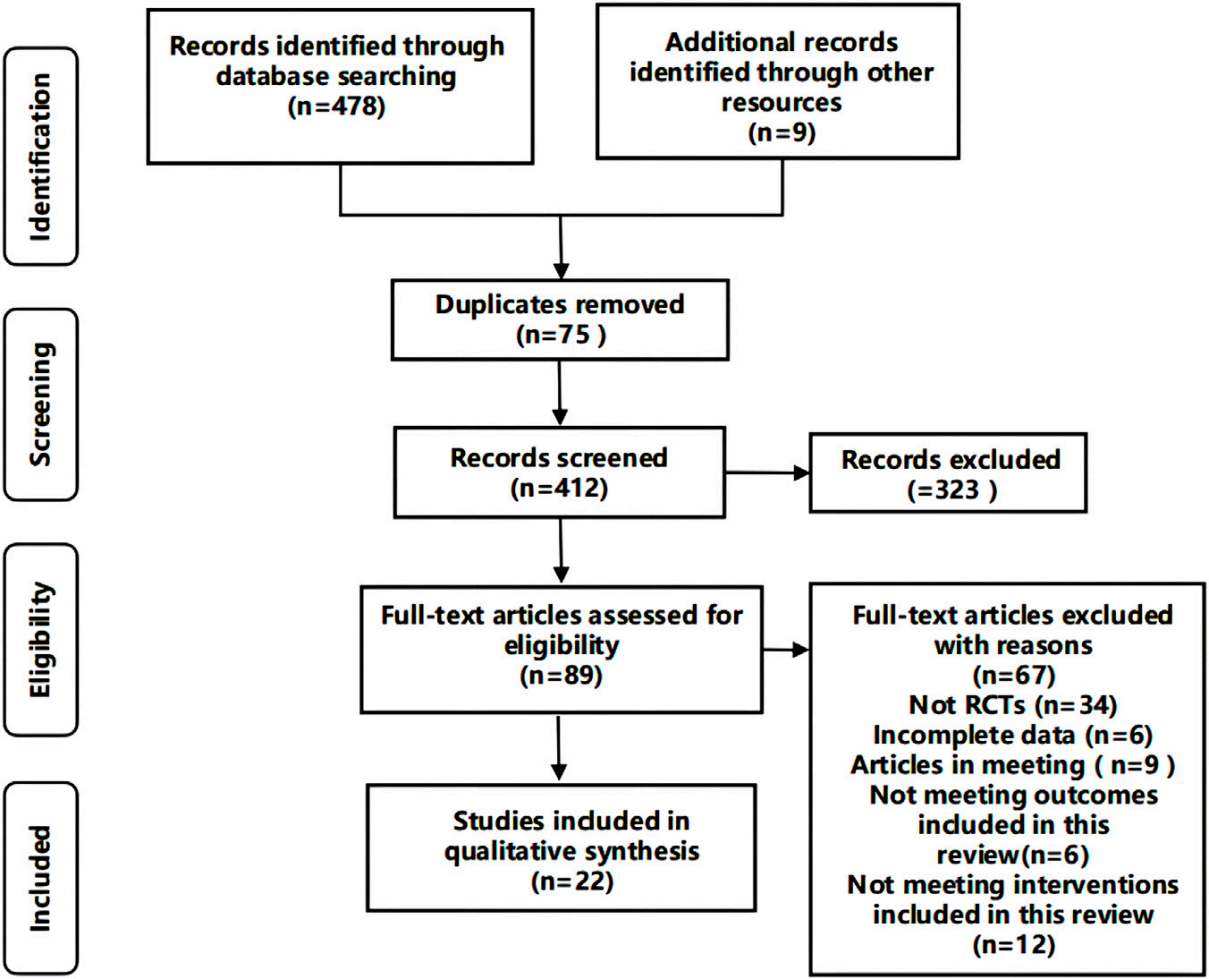
Flow diagram of literature selection.

From the electronic database, we retrieved 478 articles. Additionally, nine articles were identified through manual searches. After deduplication, we conducted title and abstract screening on 412 articles. From these, we excluded 323 due to irrelevance. A comprehensive full-text review of the 89 remaining articles resulted in the exclusion of 67 for various reasons: they weren’t randomized controlled trials, they had incomplete datasets, were only conference papers, or didn’t match our review’s intervention criteria. Finally, we selected 22 articles, comprising 4 clinical trials (Chang et al., 2016; Nejati et al., 2022; Wu et al., 2019; Yan et al., 2015). and 18 preclinical studies (Chang et al., 2010; Chen et al., 2021; Deng et al., 2019; Feng et al., 2018; Guo et al., 2016; He et al., 2016; Li et al., 2017; Lu et al., 2020; Wang et al., 2020; Wang et al., 2023; Xing et al., 2011; Xu et al., 2019; Yang et al., 2011; Yang et al., 2022; Yuan et al., 2015; Zhang et al., 2019; Zhao et al., 2017; Zhu et al., 2019) (Figure 1).

### 3.2 Description of included trials

Most studies, mainly conducted in China, had intervention durations that varied, with the majority lasting for 16 weeks. The trials by Nejati et al. (2022), Chang et al. (2016), and Yan et al. (2015) Nejati et al. adopted a treatment-control design. In contrast, the study by Wu et al. (2019) employed a self-control approach for a period of over a month. Interventions either combined berberine with lifestyle modifications or were solely focused on berberine, while control groups predominantly underwent lifestyle interventions. Each trial ensured balanced gender distribution. Further details of the preclinical studies are provided in Table 2, and additional information on the clinical studies is available in Table 3.

**TABLE 2:** The characteristics of the included preclinical studies.

Author	Country	Year	Sample size (T/C)	Weight (g)	Intervention	Control	Outcome
Chang	China	2010	8/8	T: 472.8 ± 12.4 C: 525.5 ± 10.1	Length of intervention: 16 weeks T: 200 mg/kg/day BBR	equal volume of vehicle, 0.5% methylcellulose	1, 3, 4, 8, 9, 11
Zhao	China	2017	6/6	T: 641.67 ± 35.90 C: 648 ± 69.95	Length of intervention: 32 weeks T: 150 mg/kg body weight/day BBR	equal volume of normal saline	1, 3, 4, 11, 12, 13
Chen	China	2021	10/10	-	Length of intervention: 16 weeks T: 100 mg/kg/day of BBR in 0.5% carmellose sodium	vehicle control, 4 mL/kg/day of 0.5% carmellose sodium	1, 4, 8, 9, 10, 12
Li	China	2017	8/8	T: 428.60 ± 37.37 C: 485.24 ± 50.15	Length of intervention: 12 weeks T: 150 mg/kg/day BBR	equal amount of normal saline	1, 3, 4, 8, 9, 10, 11
Xing	China	2011	10/10	T: 631.2 ± 90.9 C: 683.7 ± 68.9	Length of intervention: 12 weeks T: 187.5 mg/kg/day BBR	3 mL/kg/day saline	1, 3, 4, 8, 9, 10, 11, 12, 13
Yang	China	2011	10/10	-	Length of intervention: 12 weeks T: 324 mg/kg BBR	distilled water	1, 3, 4, 10
Zhang	China	2019	16/16	-	Length of intervention: 16 weeks T: 100 mg/kg/day BBR	equal distilled water/day	1, 2, 3, 4, 5, 8, 9, 10
Wang	China	2023	8/8	-	Length of intervention: 12 weeks T: 200 mg/kg/day BBR	equal volume of 0.5% methylcellulose	1, 3, 4, 5, 6, 7, 8, 9, 12, 10, 14, 15
He	China	2016	10/10	-	Length of intervention: 8 weeks T: 50 mg/kg/day BBR	intraperitoneal injection of saline	3, 10
Zhu	China	2019	3–6/3–6	-	Length of intervention: 12 weeks T: 300 mg/kg/day BBR	0.5% carmellose sodium	5, 7
Xu	China	2019	10/7–8	T: 36.82 ± 1.63 C: 42.60 ± 1.56	Length of intervention: 12 weeks T: 300 mg/kg/d BBR	equal volume of 0.5% methylcellulose	1, 3, 4, 6, 7, 11, 12, 10
Lu	China	2020	8/8	-	Length of intervention: 12 weeks T: not provided BBR dose	high-fat diet	2, 4, 5, 8, 9, 16
Yuan	China	2015	8/8	-	Length of intervention: 24 weeks T: 200 mg/kg/day BBR	high-fat diet	1, 3, 5
Deng	China	2009	8/8	-	Length of intervention: 16 weeks T: 100 mg/kg body weight/day BBR	10 mL/kg body weight distilled water	1, 3, 4, 6, 7, 10, 14, 15
Feng	China	2018	12/12	T: 508.04 ± 18.14 C:518.22 ± 42.26	Length of intervention: 12 weeks T: 100 mg/kg bw BBR	0.5% sodium carboxymethylcellulose	1, 4, 8, 9, 11, 10, 16
Yang	China	2022	35/35	-	Length of intervention: 10 weeks T: 40 mg/kg, BBR	not provided	1, 3, 4, 6, 7, 8, 9, 10, 14
Guo	China	2016	4–7/4–7	-	Length of intervention: 12 weeks T: 100 mg/kg body weight/day BBR	low-fat diet	14
Wang	China	2020	12/12	-	Length of intervention: 12 weeks T: 200 mg/kg/day BBR	10 mL/kg/day distilled water	1, 3, 4, 8, 9, 10

Note: 1: TC; 2: Hepatic TC; 3: LDL; 4: TG; 5: Hepatic TG; 6: liver TC; 7: Liver TG; 8: ALT; 9: AST; 10: HDL; 11: Body weight; 12: fasting blood glucose; 13: fasting insulin; 14: Liver weight; 15: Liver-to-body ratio; 16: Free fatty acids.

**TABLE 3:** The characteristics of the included clinical studies.

Author	Country	Year	Population	Age (years)	Total/M/F	Intervention	Control	Outcome
Nejati	Iran	2022	NAFLD	T: 40.6 ± 8.8 C:42.2 ± 3.8	T: 24/20/4 C: 24/17/7	Length of intervention: 7 weeks T: 6.25 g/day BBR	no taking BBR	1, 2, 3, 4, 5, 6
Chang	China	2016	NAFLD	T: 51.2 ± 9.4 C: 50.8 ± 10.4	T: 41/26/15 C: 39/20/19	Length of intervention: 16 weeks T: lifestyle intervention plus BBR (0.5g, tid)	lifestyle intervention	1, 2, 3, 4, 5, 6, 7
Wu	China	2019	NAFLD	-	-	Length of intervention: 1 month Dose: not provided Before treatment and after (self control)	before treatment	2, 7
Yan	China	2015	NAFLD	T: 50.72 ± 9.76 C:50.64 ± 10.69	T: 62/38/24 C: 62/32/30	Length of intervention: 16 weeks T: lifestyle intervention plus BBR 0.5 g tid	lifestyle intervention	1, 2, 3, 4, 5, 6

Note: 1: Body weight; 2: BMI; 3: TG; 4: TC; 5: HDL; 6: LDL; 7: fasting blood glucose.

### 3.3 Risk of bias of included studies

A summarized risk of bias for the included preclinical studies is provided in Table 4, and for the included clinical studies in Table 5. The risk of bias and limitations of each study are shown in Table 6. All studies demonstrated consistent transparency in data reporting. However, significant variations were evident in areas such as blinding and sequence generation. Notably, the study by Wu et al. (2019) stood out for its meticulous methodology.

**TABLE 4:** Summary of risk of bias assessments for included preclinical studies.

Author, year	Selection bias			Performance bias		Detection bias		Attrition bias	Reporting bias	Other	Total score
	Sequence generation	Baseline characteristics	Allocation concealment	Random housing	Blinding	Random outcome assessment	Blinding	Incomplete outcome data	Selective outcome reporting	Other sources of bias	
Chang et al. (2010)	Unclear	Unclear	Unclear	Yes	Unclear	Unclear	Unclear	Yes	Yes	Yes	14
Zhao et al. (2017)	Unclear	Unclear	Unclear	Yes	Unclear	Unclear	Unclear	Yes	Yes	Yes	14
Chen et al. (2021)	Unclear	Unclear	Unclear	Yes	Unclear	Unclear	Unclear	Yes	Yes	No	12
Li et al. (2017)	Unclear	Unclear	Unclear	Yes	Unclear	Unclear	Unclear	Yes	Yes	No	12
Xing et al. (2011)	Unclear	Unclear	Unclear	Yes	Unclear	Unclear	Unclear	Yes	Yes	Yes	14
Yang et al. (2011)	Unclear	Unclear	Unclear	Yes	Unclear	Unclear	Unclear	Yes	Yes	Unclear	13
Zhang et al. (2019)	Unclear	Unclear	Unclear	Yes	Unclear	Unclear	Unclear	Yes	Yes	Yes	14
Wang et al. (2023)	Unclear	Unclear	Unclear	Yes	Unclear	Unclear	Unclear	Yes	Yes	Yes	14
He et al. (2016)	Unclear	Unclear	Unclear	Yes	Unclear	Unclear	Unclear	Yes	Yes	Yes	14
Zhu et al. (2019)	Unclear	Unclear	Unclear	Yes	Unclear	Unclear	Unclear	Yes	Yes	Yes	14
Xu et al. (2019)	Unclear	Unclear	Unclear	Yes	Unclear	Unclear	Unclear	No	No	Yes	10
Lu et al. (2020)	Unclear	Unclear	Unclear	Yes	Unclear	Unclear	Unclear	Yes	Yes	Yes	14
Yuan et al. (2015)	Unclear	Unclear	Unclear	Yes	Unclear	Unclear	Unclear	Yes	Yes	Yes	14
Deng et al. (2019)	Unclear	Unclear	Unclear	Yes	Unclear	Unclear	Unclear	Yes	Yes	Yes	14
Feng et al. (2018)	Unclear	Unclear	Unclear	Yes	Unclear	Unclear	Unclear	Yes	Yes	Yes	14
Yang et al. (2022)	Unclear	Unclear	Unclear	Yes	Unclear	Unclear	Unclear	Yes	Yes	Yes	14
Guo et al. (2016)	Unclear	Unclear	Unclear	Yes	Unclear	Unclear	Unclear	No	No	Yes	10
Wang et al. (2020)	Unclear	Unclear	Unclear	Yes	Unclear	Unclear	Unclear	Yes	Yes	Yes	14

**TABLE 5:** Summary of risk of bias assessments for included clinical studies.

Cochrane-RCT	Random sequence generation	Allocation concealment	Blinding of participants and personnel	Blinding of outcome assessment	Incomplete outcome data	Selective reporting	Other bias
Nejati et al. (2022)	Unclear	Unclear	Unclear	Unclear	Yes	Yes	Yes
Chang et al. (2016)	Unclear	Unclear	Unclear	Unclear	Yes	Yes	Yes
Yan et al. (2015)	Unclear	Unclear	Unclear	Unclear	Yes	Yes	Yes
ROBINS-I	Confounding	Selection bias	Bias in measurement classification of interventions	Bias due to deviations from intended interventions	Bias due to missing data	Bias in measurement of outcomes	Bias in selection of the reported result
Wu et al. (2019)	Yes	Yes	Yes	Yes	Yes	Yes	Yes

**TABLE 6:** Risk of bias and limitations of each study.

Author, year	Risk of bias level	Limitations
Chang et al. (2010)	Low	Lack of blinding and sequence generation details
Zhao et al. (2017)	Low	Lack of blinding and sequence generation details
Chen et al. (2021)	Low	Lack of blinding and sequence generation details
Li et al. (2017)	Low	Lack of blinding and sequence generation details
Xing et al. (2011)	Low	Lack of blinding and sequence generation details
Yang et al. (2011)	Low	Unclear reporting bias
Zhang et al. (2019)	Low	Lack of blinding and sequence generation details
Wang et al. (2023)	Low	Lack of blinding and sequence generation details
He et al. (2016)	Low	Lack of blinding and sequence generation details
Zhu et al. (2019)	Low	Lack of blinding and sequence generation details
Xu et al. (2019)	Low	Lack of blinding and sequence generation details
Lu et al. (2020)	Low	Lack of blinding and sequence generation details
Yuan et al. (2015)	Low	Lack of blinding and sequence generation details
Deng et al. (2009)	Low	Lack of blinding and sequence generation details
Feng et al. (2018)	Low	Lack of blinding and sequence generation details
Yang et al. (2022)	Low	Lack of blinding and sequence generation details
Guo et al. (2016)	Low	Lack of blinding and sequence generation details
Wang et al. (2020)	Low	Lack of blinding and sequence generation details
Nejati et al. (2022)	Moderate	Unclear sequence generation and allocation concealment
Chang et al. (2016)	Moderate	Unclear sequence generation and allocation concealment
Yan et al. (2015)	Moderate	Unclear sequence generation and allocation concealment
Wu et al. (2019)	Low	Detailed methodology, but potential for selection bias

### 3.4 Sequence generation and allocation concealment

Ambiguity was evident regarding sequence generation and allocation concealment in studies by Nejati et al. (2022), Chang et al. (2016), Yan et al. (2015). These inconsistencies raise concerns about the randomness and fidelity of allocations in these trials. In contrast, the study by Wu et al. (2019) transparently addressed both random sequence generation and allocation concealment.

### 3.5 Blinding, incomplete outcome data, and selective reporting

Blinding, a crucial aspect for reducing biases, was unclear in the studies by Nejati et al. (2022), Chang et al. (2016), Yan et al. (2015). This inconsistency raises a concern. However, the study by Wu et al. (2019) distinctly confirmed the blinding of both participants and outcome assessments. Additionally, all of the aforementioned studies confirmed the complete reporting of outcome data without any signs of selective reporting.

### 3.6 Additional potential biases

All RCTs, including Wu et al. (2019), indicated the existence of other potential biases, though the specifics were not detailed. The universal presence of these biases suggests caution in interpretation and indicates factors that might not be covered in typical evaluations.

### 3.7 Clinical efficacy of berberine in NAFLD patients

Both LDL cholesterol and TC exhibited notable heterogeneity in the examined studies. Specifically, LDL cholesterol had an I^2^ value of 70.3% with a significant P-value of 0.034. TC displayed an I^2^ of 80.3% with a P-value of 0.006, highlighting the substantial variability among the included studies for these markers. Conversely, the heterogeneity for TG was relatively moderate with an I^2^ value of 50.9%, indicating more consistency in the results for this parameter.

Interestingly, despite the minimal heterogeneity observed for HDL cholesterol, weight, and BMI, the effect sizes (SMD) were rather small, registering at −0.09, 0.10, and 0.05 respectively. This suggests that while the studies were consistent in their findings for these parameters, berberine treatment did not result in significant changes in these metrics.

**FIGURE 2:**
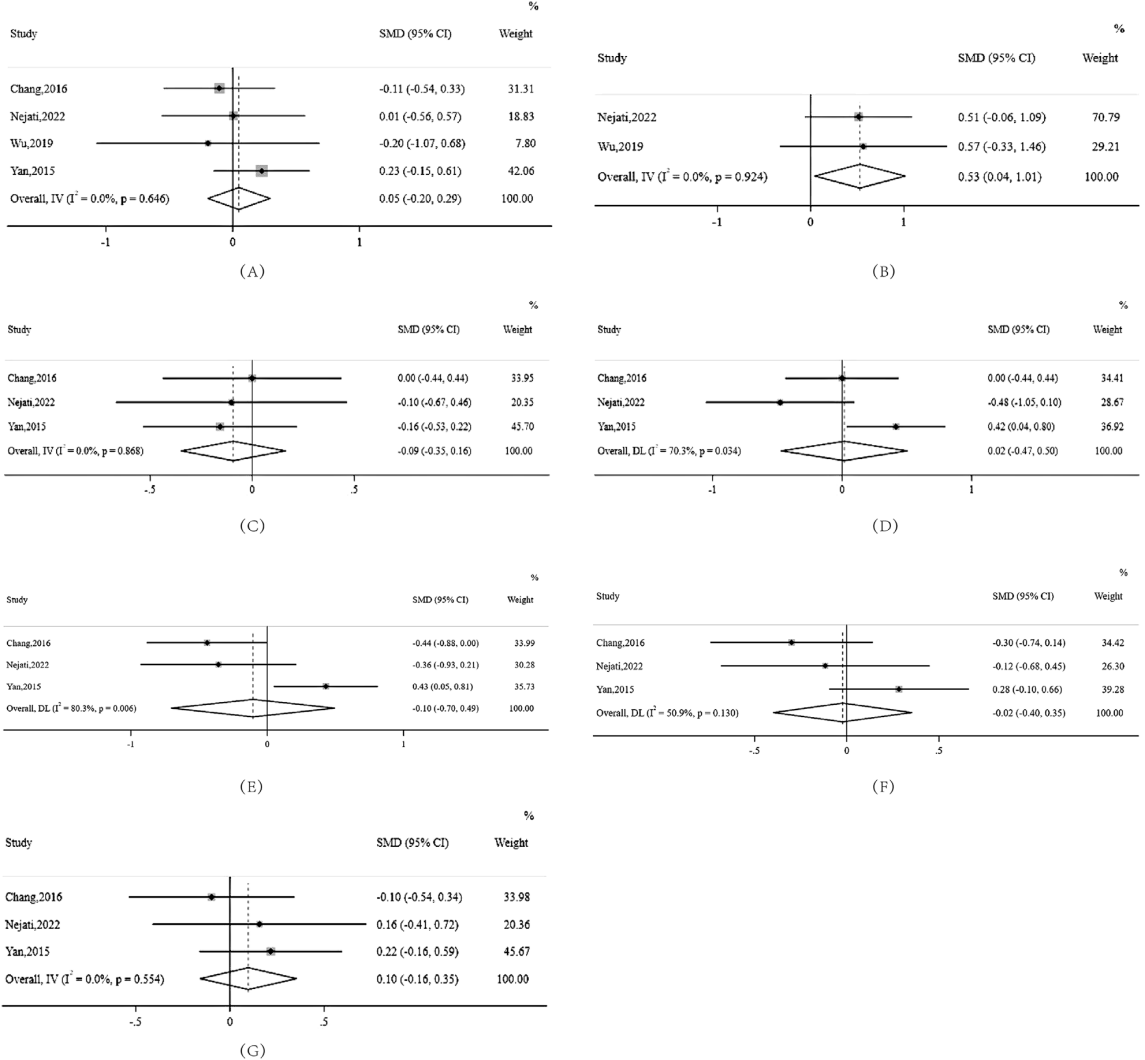
Forest plots for various health markers in NAFLD patients treated with berberine **(A)** BMI **(B)** FBG **(C)** HDL **(D)** LDL **(E)** TC **(F)** TG **(G)** Weight. Note:IV:inverted variance SMD:standardised mean difference DL: derSimonian-laird.

Most remarkably, berberine showed strong potential for positively modulating FBG levels. The effect size (SMD) of FBG was 0.53, with a 95% confidence interval ranging from 0.04 to 1.01. This indicates the promising therapeutic effect of berberine on fasting blood glucose, which could have implications for its potential use in glucose metabolism disorders or conditions like diabetes (Figure 2).

### 3.8 Sensitivity analysis

To robustly ascertain the stability of our meta-analytic results on TC, we meticulously performed a detailed sensitivity analysis, omitting each study sequentially and recalculating the combined effect size. Upon omitting the study by Chang et al. (2016), the recalibrated effect size was found to be 0.349. Upon the exclusion of the study by Nejati et al. (2022), the effect size shifted slightly to 0.192. Notably, there was a substantial change in the direction of the effect size, measuring at −0.405, upon omission of the study by Yan et al. (2015). This marked variability underscores the significant influence of Yan’s study (Yan et al., 2015) in shaping the comprehensive meta-analytic outcome for TC. (Supplementary Figure S1)

### 3.9 Preclinical influence of berberine on NAFLD models

The majority of indicators, such as ALT, AST, FBG, FFA, HDL, LDL, Hepatic TG, liver TC, liver TG, TC, TG, and weight, showed significant effect sizes. These results suggest that berberine intervention has a positive impact on these markers. Furthermore, we noted that the 95% CI for most of these indicators did not encompass 0, indicating that these effects are statistically significant. However, for certain markers, such as FINS and Hepatic TC, the confidence intervals spanned 0, implying a lower certainty in these effects.

#### 3.9.1 Liver function indicators

Primarily, from the perspective of hepatic injury, ALT and AST, which serve as pivotal indicators of hepatocellular damage, exhibited pronounced reductions across.

**FIGURE 3:**
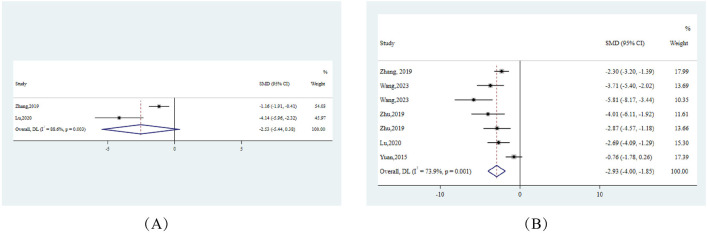
Forest plots for liver function indicators in response to berberine treatment **(A)** ALT **(B)** AST. Note:SMD:standardised mean difference.

10 experiments. Specifically, ALT showed an effect size (SMD) of −2.74 (95% CI: −4.00, −1.49), and AST reported an SMD of −2.53 (95% CI: −3.75, −1.31). Both markers displayed substantial heterogeneity with I^2^ values exceeding 90% (Figure 3).

#### 3.9.2 Glycemic and lipid metabolic response

**FIGURE 4:**
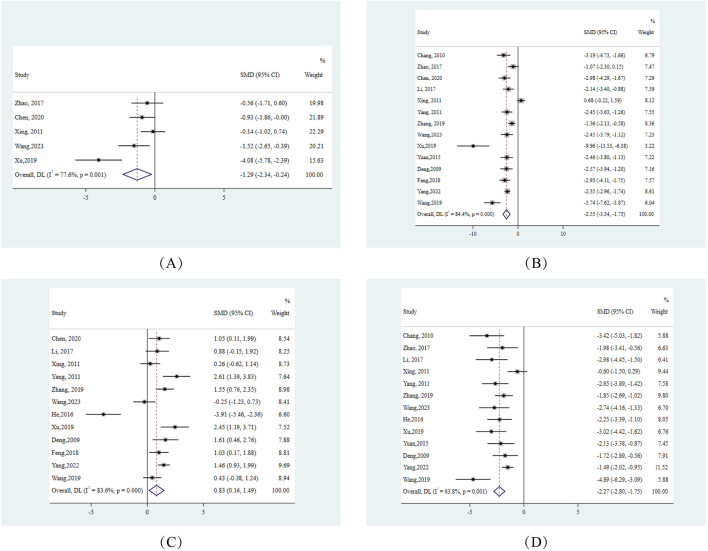
Forest plots for glycemic and lipid metabolic responses to berberine treatment **(A)** HDL **(B)** LDL **(C)** FBG **(D)** TC. Note:SMD:standardised mean difference DL:derSimonian-laird.

On the metabolic front, FBG levels from 5 studies manifested a notable decline, highlighted by an SMD of −1.29 (95% CI: −2.34, −0.24), highlighting berberine’s positive modulation of glucose metabolism. Concurrently, we observed lipid metabolism alterations: HDL levels surged in 12 studies, demonstrated by an SMD of 0.83 (95% CI: 0.16, 1.49). In contrast, LDL and TC levels decreased across 13 and 14 studies respectively, with effect sizes measuring −2.27 (95% CI: −2.80, −1.75) for LDL and −2.55 (95% CI: −3.34, −1.75) for TC (Figure 4).

#### 3.9.3 Hepatic lipid accumulation insights

**FIGURE 5:**
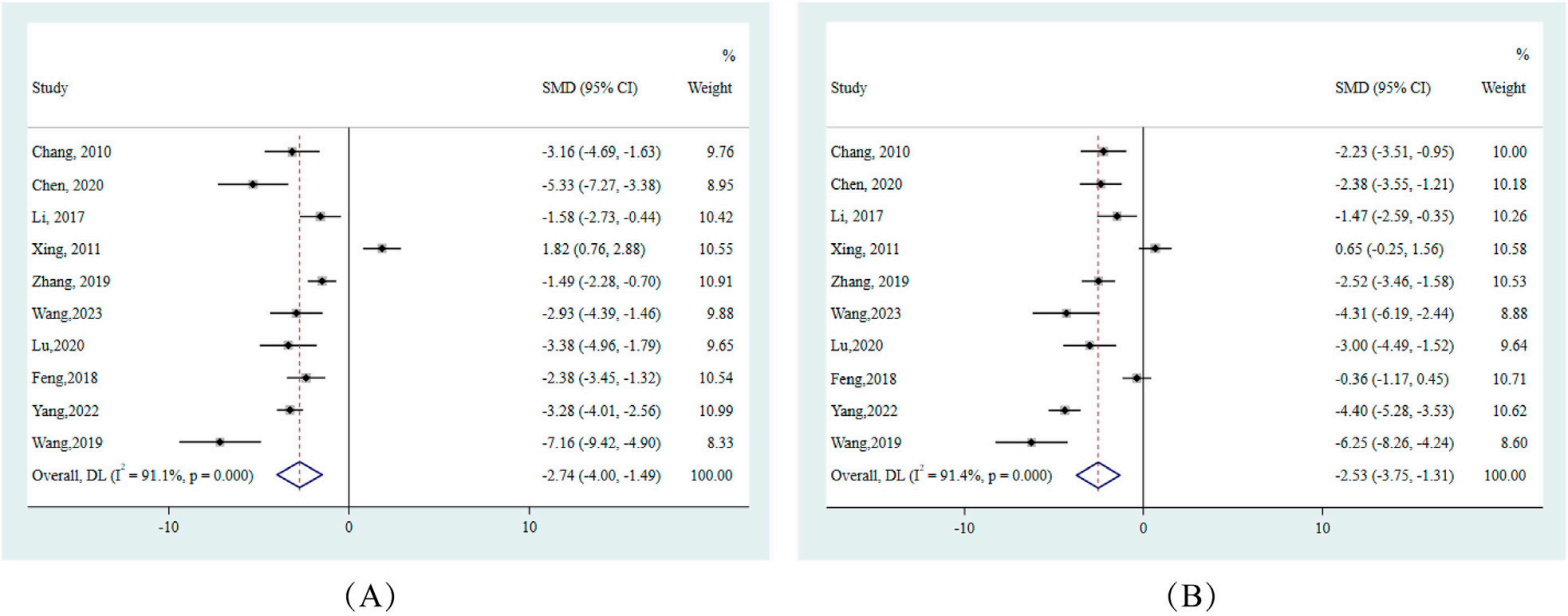
Forest plots for hepatic lipid accumulation indicators in response to berberine treatment **(A)** Hepatic TC **(B)** Hepatic TG. Note:SMD:standardised mean difference DL:derSimonian-laird.

Delving deeper into the quintessence of NAFLD, which is hepatic lipid accumulation, we discerned significant reductions in hepatic TC and TG levels. Notably, hepatic TG across 7 studies demonstrated an SMD of −2.93 (95% CI: −4.00, −1.85), indicating berberine’s potential in curtailing intracellular hepatic fat deposition (Figure 5).

### 3.10 Subgroup of main result

Subgroup analysis demonstrated that berberine exhibited significant dose-and treatment-dependent effects on key biochemical markers of NAFLD. Regarding dosage, the ≤100 mg/kg group showed the most pronounced reduction in AST (SMD −2.41; 95% CI −4.21 to −0.61) and ALT (−2.92; −4.17 to −1.66), with controlled heterogeneity (I^2^ = 0–84%). In contrast, the >150 mg/kg group exhibited deeper point estimates but wider confidence intervals and higher heterogeneity (I^2^ > 94%), suggesting potential interstudy differences or nonlinear responses from excessive doses. LDL and total cholesterol showed robust reductions at intermediate-to-high doses (100–150 mg/kg and >150 mg/kg), with low-to-moderate heterogeneity (I^2^ = 0–77%). For duration, AST demonstrated significant improvement only within ≤12 weeks (−2.59; P < 0.001), while AST, LDL, and TC showed statistically significant decreases in both ≤12 and >12 weeks. Notably, HDL elevation achieved statistical significance only in the >12-week subgroup (1.40; P < 0.001) with zero heterogeneity. Taken together, a dose of around 100 mg/kg for at least 12 weeks may be the optimal window for both efficacy and interpretability of heterogeneity. (Detailed results are shown in Table 5).

## 4 Discussion

### 4.1 Main outcomes summary

This systematic review and meta-analysis aimed to evaluate the effectiveness of berberine in the treatment of NAFLD. We conducted a thorough literature search across five major databases, identifying four clinical trials and eighteen preclinical studies that met our inclusion criteria. Our meta-analysis demonstrates that berberine significantly modulates key biochemical markers, including TC, TG, LDL, ALT, AST, and body weight, across studies in both preclinical and human settings. Additionally, we observed a favorable impact on HDL levels in preclinical settings. However, the effects on FINS and hepatic TC were modest. In clinical trials, berberine resulted in a notable reduction in FBG levels. Nonetheless, certain parameters like liver weight and liver-to-body ratio in preclinical studies, and BMI in clinical trials, yielded results that were either inconsistent or inconclusive.

### 4.2 Applicability of evidences

To understand these metabolic and physiological changes, it is crucial to explore the molecular mechanisms through which berberine acts. One major area of influence is hepatic lipid metabolism (Li S. et al., 2020). Berberine has garnered attention due to its ability to modulate energy homeostasis by activating AMP-activated protein kinase (AMPK), which is a pivotal orchestrator of cellular energy metabolism. Once activated, AMPK exerts inhibitory effects on sterol regulatory element-binding protein 1c (SREBP-1c), which is a key transcriptional regulator of lipid synthesis (Feng et al., 2019; Pei et al., 2019; Xu et al., 2021). The consequential downregulation of specific enzymes associated with SREBP-1c, such as the pivotal stearoyl-CoA desaturase-1, results in the inhibition of lipid synthesis, leading to reduced levels of triglycerides in the liver. These reductions subsequently influence liver weight and the liver-to-body ratio, highlighting berberine’s potential therapeutic implications in lipid-related disorders (Ge et al., 2011; Hu et al., 2010). Additionally, berberine affects various signaling pathways involved in hepatic steatosis (Song et al., 2020). Activation of peroxisome proliferator-activated receptor alpha (PPAR-α) aids in breaking down FFA in the liver (Qin et al., 2019; Zhong et al., 2022). In preclinical studies, the activation of specific metabolic pathways by berberine has been demonstrated to exert a notable influence on FFA concentrations. Notably, the inhibition of fatty acid synthase, an enzyme pivotal to lipid synthesis, leads to a marked reduction in lipid accumulation within cells. Furthermore, berberine’s interaction with the liver X receptor, a nuclear receptor involved in lipid metabolism and inflammation, underscores its multifaceted role in lipid homeostasis (Luo et al., 2016).

Berberine’s role extends to cholesterol regulation. It reduces the activity of the enzyme 3-hydroxy-3-methyl-glutaryl-CoA reductase, which leads to decreased levels of TC and LDL (Cao et al., 2013; Chang et al., 2012). Berberine-mediated augmentation of PPAR-α activity could also potentially drive an elevation in HDL concentrations (Ferri et al., 2017). Concerning elevated AST and ALT levels, berberine activates AMPK and inhibits pro-inflammatory pathways such as the Nuclear Factor kappa-light-chain-enhancer of activated B cells, potentially reducing liver inflammation (Di Cara et al., 2023; Huby and Gautier, 2022).

In the liver, berberine inhibits gluconeogenesis by downregulating key enzymes such as phosphoenolpyruvate carboxykinase and glucose-6-phosphatase, effectively lowering FBG levels (Jiang et al., 2015; Zhang et al., 2018). Concurrently, it enhances insulin sensitivity by facilitating the phosphorylation of insulin receptor substrate-1 and promoting the translocation of glucose transporter type 4 in muscle cells, thereby further contributing to glucose regulation (Hu et al., 2022; Xia et al., 2011).

The minimal impact of berberine on FINS is primarily due to its mode of action, which focuses on improving insulin resistance rather than directly stimulating the pancreas to secrete additional insulin. Furthermore, berberine may also enhance glucose metabolism by facilitating glucose uptake in muscle and liver cells, thus reducing the immediate need for an elevation in FINS levels (Ilyas et al., 2020; Li W. et al., 2020).

### 4.3 The strengths and limitation of this review

In this study, we conducted a systematic review and meta-analysis focusing on berberine’s impact on NAFLD. To our knowledge, this study is one of the few investigations that combine both clinical and preclinical data, thereby deepening our understanding. By adhering to the rigorous standards of the Cochrane Handbook for Systematic Reviews of Interventions, our methodology enhances the reliability of our results. While we anticipate that our findings will strengthen the case for berberine as an evidence-based treatment for NAFLD, it’s important to note that further research is essential to confirm these initial observations.

While our study provides promising results regarding the efficacy of berberine in treating NAFLD, several limitations must be considered when extrapolating these findings to clinical practice. Preclinical studies, often conducted in controlled environments or using animal models, may not fully capture the complexity of human disease, including genetic variability, environmental factors, and comorbidities. Translating preclinical mechanisms to human outcomes is challenging, as species-specific differences in metabolic pathways could mean that effects observed in rodents, such as AMPK activation and lipid metabolism, may not be directly applicable to humans. The duration and dosage of berberine used in preclinical studies may also not be suitable for clinical settings.Additionally, small sample sizes in some studies may limit the statistical power and generalizability of the findings.

A key limitation is the potential for confounding biases in clinical studies. Concurrent medications, such as statins and antidiabetic drugs, which independently improve NAFLD outcomes, could skew results if not accounted for. Animal studies often use berberine doses of 100–200 mg/kg (converted by body surface area, equivalent to 8–16 mg/kg in humans), while the commonly used clinical dose is 0.9–1.5 g/day (≈12–21 mg/kg). The dose difference may partly explain the phenomenon that the clinical effect size is lower than that in animal experiments.Future studies should collect comprehensive medication histories and adjust for potential confounders to accurately assess berberine’s independent effects.

Furthermore, our review focused on biochemical changes associated with NAFLD, rather than directly assessing the impact of berberine on fatty liver changes. Future research should incorporate specific diagnostic methods, such as liver biopsy, SWE/CAP ultrasound, or chemical shift-encoded MRI, to provide a more accurate assessment of berberine’s effects on hepatic fat burden.

Despite these limitations, the findings from our study suggest potential clinical applications for berberine in the management of NAFLD. The observed improvements in biochemical markers and liver function suggest that berberine may play a role in reducing liver inflammation and improving metabolic profiles associated with NAFLD. Future clinical trials should aim to address the limitations of previous studies by employing larger sample sizes, longer follow-up periods, and more rigorous study designs to confirm the therapeutic potential of berberine in NAFLD.

## 5 Conclusion

In our study, we found berberine effective in targeting key markers of NAFLD. Particularly in managing liver lipid metabolism and glucose balance, berberine shows potential. Overall, considering its benefits on cholesterol, glucose, and liver enzymes, berberine is recommended for individuals with NAFLD seeking improved liver health.

## Data Availability

The original contributions presented in the study are included in the article/Supplementary Material, further inquiries can be directed to the corresponding authors.
