# Supplementary material for: Biochemical changes associated with non-alcoholic fatty liver disease in response to berberine treatment: a systematic review and meta-analysis of clinical and preclinical research
Source: Front Pharmacol. 2025 Aug 29;16:1460643. doi: 10.3389/fphar.2025.1460643 (PMC12433188; doi:10.3389/fphar.2025.1460643)
Supplement: Supplementary file 1 [file DataSheet1.doc]

Supplement Figure1 Sensitivity analysis to TC
